# Bactericidal synergism between phage endolysin Ply2660 and cathelicidin LL-37 against vancomycin-resistant *Enterococcus faecalis* biofilms

**DOI:** 10.1038/s41522-023-00385-5

**Published:** 2023-04-06

**Authors:** Huihui Zhang, Xinyuan Zhang, Siyu Liang, Jing Wang, Yao Zhu, Wanjiang Zhang, Siguo Liu, Stefan Schwarz, Fang Xie

**Affiliations:** 1 State Key Laboratory for Animal Disease Control and Prevention, Harbin Veterinary Research Institute, Chinese Academy of Agricultural Sciences, Harbin, China; 2 Institute of Microbiology and Epizootics, Centre for Infection Medicine, School of Veterinary Medicine, Freie Universität Berlin, Berlin, Germany; 3 Veterinary Centre for Resistance Research (TZR), Freie Universität Berlin, 14163 Berlin, Germany

**Keywords:** Antimicrobials, Bacteria, Bacteriology, Biofilms

## Abstract

Antibiotic resistance and the ability to form biofilms of *Enterococcus faecalis* have compromised the choice of therapeutic options, which triggered the search for new therapeutic strategies, such as the use of phage endolysins and antimicrobial peptides. However, few studies have addressed the synergistic relationship between these two promising options. Here, we investigated the combination of the phage endolysin Ply2660 and the antimicrobial peptide LL-37 to target drug-resistant biofilm-producing *E. faecalis*. In vitro bactericidal assays were used to demonstrate the efficacy of the Ply2660–LL-37 combination against *E. faecalis*. Larger reductions in viable cell counts were observed when Ply2660 and LL-37 were applied together than after individual treatment with either substance. Transmission electron microscopy revealed that the Ply2660–LL-37 combination could lead to severe cell lysis of *E. faecalis*. The mode of action of the Ply2660–LL-37 combination against *E. faecalis* was that Ply2660 degrades cell wall peptidoglycan, and subsequently, LL-37 destroys the cytoplasmic membrane. Furthermore, Ply2660 and LL-37 act synergistically to inhibit the biofilm formation of *E. faecalis*. The Ply2660–LL-37 combination also showed a synergistic effect for the treatment of established biofilm, as biofilm killing with this combination was superior to each substance alone. In a murine peritoneal septicemia model, the Ply2660–LL-37 combination distinctly suppressed the dissemination of *E. faecalis* isolates and attenuated organ injury, being more effective than each treatment alone. Altogether, our findings indicate that the combination of a phage endolysin and an antimicrobial peptide may be a potential antimicrobial strategy for combating *E. faecalis*.

## Introduction

*Enterococcus faecalis* is an opportunistic pathogen that has emerged as a major cause of healthcare-associated infections with high mortality rates^1^. This Gram-positive bacterium is able to survive under harsh conditions, making it well adapted to the hospital environment^2,3^. In addition, the worldwide spread of multidrug-resistant isolates, especially the emergence of vancomycin-resistant *E. faecalis*, puts huge economic pressure on the current healthcare systems because of the increasing risk of treatment failure and death^4,5^. Compared to the closely related species *Enterococcus faecium*, *E. faecalis* shows lower levels of intrinsic and acquired antibiotic resistance but is more virulent and more often isolated from hospitals^4,5^. It is considered the third-most prevalent nosocomial bacterial pathogen worldwide^6^. The U.S. Centers for Disease Control and Prevention listed vancomycin-resistant *Enterococcus* among the serious threat pathogens^7^.

Of great concern is that *E. faecalis* infections are usually linked to biofilm formation. Biofilms are dense multicellular communities of bacteria that form on biotic or abiotic surfaces, including human tissues, medical devices, and other materials^8^. Biofilm formation is a cyclic process involving initial attachment on a surface, microcolony formation, biofilm maturation, and dispersion^9,10^. Bacteria growing in the biofilm state are physiologically different from their planktonic cells, and their antibiotic tolerance can increase up to 1000-fold^11,12^. In addition, bacteria within biofilms are protected from the host defense, may persist for expanded time periods, and represent a reservoir for antibiotic resistance^13,14^. Biofilm-producing *E. faecalis* isolates occur worldwide. In Italy, biofilm production was identified among 87% of *E. faecalis* clinical isolates^10,15^. A study from the United States reported that 93% of 163 *E. faecalis* clinical and fecal isolates were classified as biofilm producers^16^. In China, 50.4 % of 113 *E. faecalis* isolates collected from urinary tract infections (UTI) produced biofilms^17^. Investigators from Japan have reported that all 352 *E. faecalis* isolates derived from UTI patients were able to form biofilms^18^. A study from Iran showed that 91 of the 95 *E. faecalis* isolates (95.8 %) produced biofilms^19^. Biofilm-producing *E. faecalis* isolates are often observed in a number of infections, including urinary tract infections, intra-abdominal and pelvic infections, catheter-related infections, surgical wounds, and endocarditis^10,13^. Thus, biofilm formation may be an important factor in the pathogenesis of *E. faecalis* infection.

The worsening antibiotic crisis and the prevalence of biofilm-producing *E. faecalis* underscore the necessity for the development of new therapeutic strategies. Antimicrobial peptides are bioactive small molecules produced by diverse organisms, which act as the first line of host defense against bacterial infections^20^. Among them, LL-37 is a 37-amino acid cationic peptide and the sole human member of the cathelicidin family^21^. This peptide is produced by many cell types, including epithelial cells, neutrophils, macrophages, and natural killer (NK) cells^22^. LL-37 displays outstanding antimicrobial activity and is active against a broad spectrum of microorganisms, including bacteria, viruses, and fungi^23^. In addition to its broad-spectrum antimicrobial activities, LL-37 has potent and multifarious immunomodulatory properties, including chemoattractant function, the release of pro-inflammatory cytokine, inhibition of neutrophil apoptosis, stimulation of angiogenesis, and tissue regeneration^22,24^. LL-37 also can augment the release of anti-inflammatory cytokines, neutralize bacterial LPS, and limit the expansion of inflammation^25,26^. Therefore, it has attracted much interest and is considered a promising agent to combat multidrug-resistant bacteria.

Another novel antibacterial strategy involves the use of phages and phage endolysins to control drug-resistant bacterial pathogens. Phages are viruses that can infect and kill bacteria. When following a lytic cycle, phages reorganize the host cell machinery to produce their own progeny. Phage endolysins are produced in phage-infected bacterial cells at the end of the phage replication cycle to destroy the cell walls and enable the release of the phage progeny^27^. Therefore, endolysins can be developed as antibacterial agents as they can cleave covalent bonds in the peptidoglycan cell wall of Gram-positive bacteria and induce cell death^28^. Compared to phages, endolysins as potential antimicrobial agents have distinct advantages. Phages usually infect specific bacteria with specificity, whereas several phage endolysins exhibit a broad lytic spectrum. For instance, PlyV12 has the capacity to kill enterococcal, streptococcal, and staphylococcal isolates^29^. In addition, endolysins are convenient for genetic engineering to improve the lytic activity^30^, expand the lytic spectrum^31^, or enhance the intracellular bactericidal activity^32^. Endolysins have better safety profiles as therapeutic agents as these enzymes exhibit non-cytotoxicity and a low probability of resistance development^33,34^. In contrast to broad-spectrum antibiotics, the antibacterial spectrum of endolysins is rather narrow, and, therefore, they do not destroy the physiological microbiota^35^. In addition, it appeared that the application of endolysins was an efficient strategy for preventing and eliminating biofilm-related infections^36,37^.

Combination therapy involving an antimicrobial peptide and a phage endolysin is considered to be a promising strategy to resolve the current clinical problem of severe antibiotic resistance in many pathogenic bacteria^38^. However, only limited data are currently available about the therapeutic efficacy of such combinations, and not all combinations are suitable for all bacteria. Thus, targeted approaches are necessary, which must be tailor-made for each bacterial pathogen. In this study, we explored the antibacterial and antibiofilm activities of the combination of the phage endolysin Ply2660, originating from a *Streptococcus suis* prophage, and the antimicrobial peptide LL-37 against *E. faecalis* in vitro, and evaluated the efficacy of this combination against vancomycin-resistant *E. faecalis* in a murine model of peritoneal septicemia.

## Results

### In vitro activity of combined Ply2660 and LL-37 against *E. faecalis*

The antimicrobial peptide LL-37 displays an α-helical amphipathic structure, with higher hydrophobic residues and a net positive charge distributed on each side (Supplementary Fig. 1a). Ply2660 has a typical organization characteristic of phage endolysins targeting Gram-positive bacteria, including an N-terminal cysteine–histidine-dependent amidohydrolase/peptidase (CHAP) catalytic domain and a C-terminal cell wall-binding domain of SH3b (Supplementary Fig. 1b). The three-dimensional (3D) structure of Ply2660 was simulated using that of phage lysin Ly7917 (PDB code 5D74) as a template. Automated molecular modeling of the 3D structure generated a homology model consisting of six α-helices and six β-sheets in the CHAP domain, and eight β-sheets in the SH3 domain (Supplementary Fig. 1c). To examine the bacteriolytic activity, full-length Ply2660 was overexpressed in *Escherichia coli* BL21 (DE3) as a soluble protein, and the recombinant protein was purified using Ni^2+^-affinity chromatography. Successful purification was confirmed by sodium dodecyl sulfate-polyacrylamide gel electrophoresis (SDS-PAGE, Supplementary Fig. 1d).

**Fig. 1:**
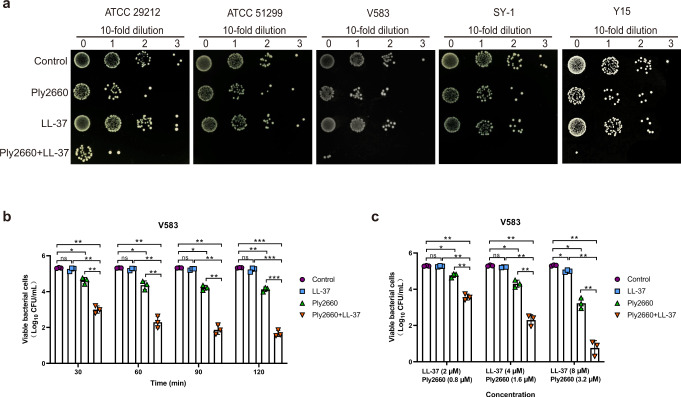
Bactericidal activity of LL-37 and/or Ply2660 against *E. faecalis* strains. **a** *Enterococcus faecalis* strains were treated with LL-37, Ply2660, or a mixture of both and incubated for 1 h at 37 °C. Tenfold serial dilutions of each sample after different treatments were spotted onto BHI agar and incubated at 37 °C for 18 h. Controls were treated with PBS alone. These assays were repeated three times on independent occasions with similar results, and representative experiments are shown. **b** Time-dependent killing efficacy of LL-37 or/and Ply2660 against *E. faecalis* V583. Bacterial cells were washed with PBS and treated with 4 μM of LL-37, 1.6 μM of Ply2660, or a mixture of both for different times (30, 60, 90, and 120 min); the viable cell number after each treatment is determined by plating on brain heart infusion (BHI) agar. **c** Dose-dependent killing efficacy of LL-37 or/and Ply2660 against *E. faecalis* V583. Bacterial cells were washed with PBS and treated with 2–8 μM of LL-37, 0.8–3.2 μM of Ply2660, or a mixture of both for 60 min, the viable cell number after each treatment is determined by plating on BHI agar. The data are presented as the means ± SD from three independent assays; error bars represent the standard deviation. Statistical significance was calculated using two-way ANOVA followed by Tukey’s multiple comparison test. *P < 0.05; **P < 0.01; ***P < 0.001; ns, not significant.

The bactericidal activities of LL-37 and/or Ply2660 against five *E. faecalis* isolates, including vancomycin-resistant and multidrug-resistant clinical isolates, were subsequently determined in vitro (Fig. 1a). No significant reduction in the number of viable bacteria was observed when LL-37 was applied at a concentration of 4 μM (Fig. 1a, b), at which it could effectively kill other bacteria, including *E. coli*, *Klebsiella pneumoniae*, *Acinetobacter baumannii*, *Pseudomonas aeruginosa*, *Staphylococcus aureus*, and *Enterococcus faecium*^39^. When evaluating the effects of Ply2660 alone in the absence of LL-37, Ply2660 at a concentration of 1.6 μM resulted in a minor reduction in the number of viable bacteria, only decreasing 0.7-log_10_ colony forming units (CFU) within 30 min. When the treating time prolonged to 120 min, Ply2660 achieved a reduction of 1.2-log_10_ CFU (Fig. 1b). Treatment with the Ply2660+LL-37 combination at the same concentration led to a decrease of 2.3-log_10_ CFU within 30 min and a 3.6-log_10_ decrease within 120 min (Fig. 1b). The concentration-kill experiments showed that 0.8 μM Ply2660 kills 0.5-log_10_ CFU of viable bacteria after treatment for 60 min, and a decrease of 2.0-log_10_ CFU of *E. faecalis* is observed when treated with 3.2 μM of Ply2660 (Fig. 1c). However, no significant reduction in the number of viable bacteria was observed when Ply2660 was applied to *E. faecium* isolates at a concentration of 3.2 μM (Supplementary Fig. 2). The Ply2660+LL-37 combination exhibited the enhanced bactericidal activity against *E. faecalis* in a dose-dependent manner (Fig. 1c). Within 60 min, the combination of Ply2660 (3.2 μM) and LL-37 (8 μM) showed high activity against *E. faecalis*, with a reduction of 4.5-log_10_ CFU (Fig. 1c). These results suggested that LL-37 can potentiate the bactericidal effect of Ply2660 on *E. faecalis*.

### Morphological changes of *E. faecalis* treated with the combination of Ply2660 and LL-37

**Fig. 2:**
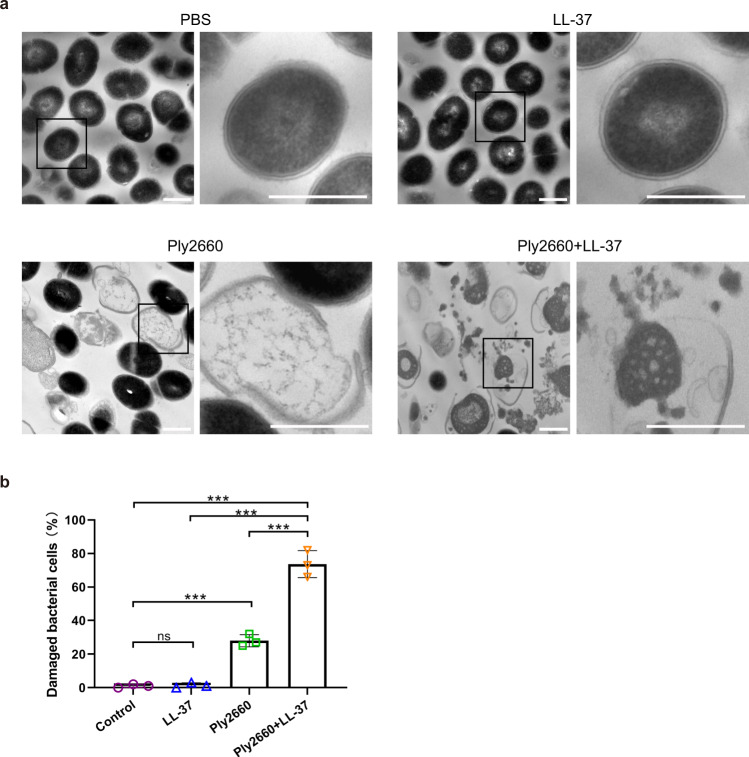
Transmission electron micrographs of *E. faecalis* after LL-37 and/or Ply2660 treatment. **a** TEM analysis of *E. faecalis* V583 in the mid-logarithmic phase or after exposure to LL-37, Ply2660, or a mixture of both was conducted. Controls were treated with PBS alone. Varying degrees of lysed morphology and leakage of contents are shown in *E. faecalis* after the different treatments. Scale bar, 500 nm. **b** Averages and standard deviations are shown for three independent counts, and the number of cells for each count was 100 (*n* = 100); error bars represent standard deviation. Statistical significance was calculated using one-way ANOVA followed by Tukey’s multiple comparison test. ***P < 0.001; ns, not significant.

To investigate the possible mechanisms underlying this synergy of Ply2660 and LL-37, the morphological changes of *E. faecalis* V583 upon exposure to Ply2660 and/or LL-37 were determined using transmission electron microscopy (TEM). As shown in Fig. 2a, untreated *E. faecalis* showed intact cells with a regular spherical shape, containing a uniform cell wall and cytoplasmic membrane and a heterogeneous electron density in the cytoplasm. LL-37 alone caused no visible morphological changes in *E. faecalis*, including cell wall and membrane ultrastructure (Fig. 2a). However, a small number of cells of *E. faecalis* treated with Ply2660 showed altered morphology with an irregular shape, a swollen and destructed appearance, and a significant decrease in electron-dense molecules in the cytoplasm (Fig. 2a). Treatment with the Ply2660+LL-37 combination led to dramatic morphological alterations of *E. faecalis*, resulting mainly in cell lysis (Fig. 2a). Most *E. faecalis* cells exhibited cytoplasmic retraction, and the intracellular content appeared granulated or clumped, or it leaked out from the ruptured sites. Considerable breakage in the cell wall and cytoplasmic membrane caused by the Ply2660+LL-37 combination could be visualized, resulting in the presence of some “ghost” cells. Moreover, a large amount of cellular debris was observed, and numerous bleb-like structures were present in the visual field. Approximately 26% of cells were damaged cells after Ply2660 treatment, whereas the Ply2660+LL-37 combination led to approximately 77% of abnormal cells (Fig. 2b). The data indicated that the loss of viability in *E. faecalis* following exposure to the combination of Ply2660 and/ LL-37 was due to their bacteriolytic properties.

### Mode of action of the combination of Ply2660 and LL-37 against *E. faecalis*

**Fig. 3:**
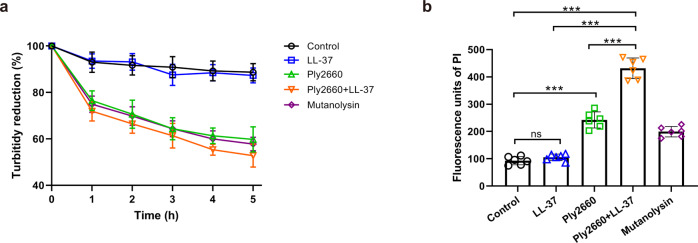
Effects of LL-37 and/or Ply2660 on cell walls and cytoplasmic membranes of *E. faecalis*. **a** Degradation of cell walls of *E. faecalis* V583 after different treatments. Cell walls were prepared from *E. faecalis* in the logarithmic growth phase and diluted with fresh PBS. LL-37, Ply2660, a mixture of both, or mutanolysin, was added into the cell wall suspension, and samples were incubated at 37 °C. Controls were treated with PBS alone. OD_600_ was measured at various time points to determine turbidity. The data are presented as the means ± SD from three independent assays; error bars represent the standard deviation. **b** Membrane damage of *E. faecalis* V583 after different treatments. *Enterococcus faecalis* was treated with LL-37, Ply2660, a mixture of both, or mutanolysin, and incubated at 37 °C for 1 h. Controls were treated with PBS alone. Membrane permeability was measured by detecting the fluorescence intensity of PI. The data are presented as the means ± SD from two independent assays with three biological replicates in each assay; error bars represent standard deviation. Statistical significance was calculated using Brown–Forsythe and Welch ANOVA tests followed by Dunnett’s T3 multiple comparison test. ***P < 0.001; ns, not significant.

To understand how LL-37 potentiates the bactericidal effect of Ply2660 on *E. faecalis*, the effects of LL-37 and/or Ply2660 on the cell wall and cytoplasmic membrane were analyzed. The purified cell wall of *E. faecalis* strain V583 was treated with LL-37, Ply2660, or a mixture of both, and the turbidity was monitored. Similar to the untreated control, LL-37 did not affect the turbidity. However, in the presence of Ply2660, the turbidity of the cell wall suspension decreased gradually as time elapsed. In addition, the Ply2660+LL-37 combination degraded the cell wall similarly to Ply2660 alone (Fig. 3a). These data indicated that Ply2660 is sufficient for the degradation of the cell wall. Subsequently, membrane damage of *E. faecalis* following exposure to Ply2660 and/or LL-37 was assessed with propidium iodide (PI), which can pass only through damaged membranes to stain nucleic acids. As shown in Fig. 3b, in the absence of Ply2660, LL-37 hardly facilitated the entry of PI into the bacteria. After the addition of Ply2660, the fluorescence intensity of PI was almost fourfold increased. This result suggested that LL-37 alone could not penetrate the cytoplasmic membrane of *E. faecalis*, unless the cell wall was degraded with the aid of Ply2660. Therefore, the outstanding bactericidal activity of the combination of Ply2660 and LL-37 is based on Ply2660-induced cell wall degradation and subsequent LL-37-mediated membrane damage.

### Ply2660 and LL-37 act synergistically against *E. faecalis* biofilm

**Fig. 4:**
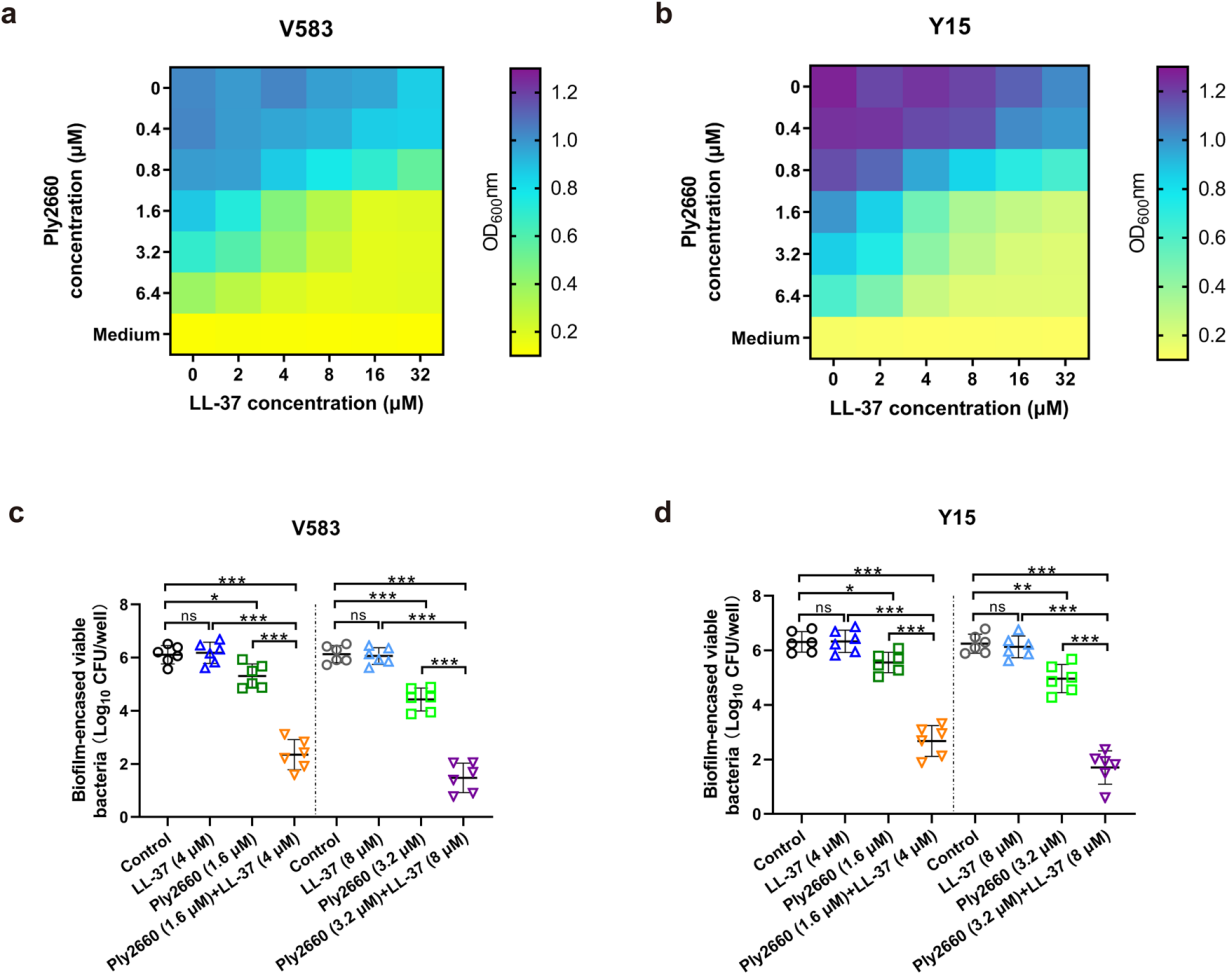
Inhibition of *E. faecalis* biofilm formation by LL-37 and/or Ply2660. Biofilm inhibition was assessed by incubating LL-37 and/or Ply2660 with *E. faecalis* isolates in the wells of 96-well polystyrene microtiter plates for 24 h. Bacteria without any treatment were used as a control. The adhered biofilms of **a** V583 and **b** Y15 isolates were measured using crystal violet staining. OD_600_ values are the average of two independent assays with three biological replicates in each assay. The number of viable bacteria in the biofilms of **c** V583 and **d** Y15 isolates was examined. Results expressed as [log_10_ CFU/well]. The data are presented as the means ± SD from two independent assays with three biological replicates in each assay; error bars represent standard deviation. Statistical significance was calculated using one-way ANOVA followed by Tukey’s multiple comparison test. *P < 0.05; **P < 0.01; ***P < 0.001; ns, not significant.

To investigate the antibiofilm activity of Ply2660 in combination with LL-37, two *E. faecalis* isolates were chosen on the basis of their ability to form biofilms. These isolates included *E. faecalis* V583, which is known as a biofilm producer^40^, and *E. faecalis* Y15, which displays an even stronger biofilm phenotype than V583 (Fig. 4a, b). In general, the minimal inhibitory concentration (MIC) value of Ply2660 against the planktonic form of *E. faecalis* isolates V583 and Y15 was 12.8 μM, and the MIC value of LL-37 was 32 μM. While the minimum biofilm inhibitory concentration (MBIC) values of Ply2660 ranged from 12.8 to 25.6 μM, and that of LL-37 was 64 μM. As shown in Fig. 4a, b, LL-37 alone did not show any inhibitory effect on the *E. faecalis* biofilm formation, except showing a minor inhibitory effect at the concentration of 32 μM. However, Ply2660 dose-dependently inhibited the biofilm at the higher concentrations (≥1.6 μM). A significant biofilm reduction was observed when Ply2660 was combined with LL-37 (Fig. 4a, b). In addition, as shown in Fig. 4c, d, LL-37 alone (at the concentrations of 4 and 8 μM) did not significantly alter the number of viable bacteria in biofilm compared to the untreated control. However, Ply2660 (at the concentrations of 1.6 and 3.2 μM) slightly reduced the number of viable bacteria (0.8 and 1.7-log_10_ CFU in V583 biofilm, 0.7 and 1.3-log_10_ CFU in Y15 biofilm). Conversely, the combination of both substances at the abovementioned concentrations led to a remarkable reduction in viable bacteria (3.7 and 4.6-log_10_ CFU in V583 biofilm, 3.6 and 4.5-log_10_ CFU in Y15 biofilm).

**Fig. 5:**
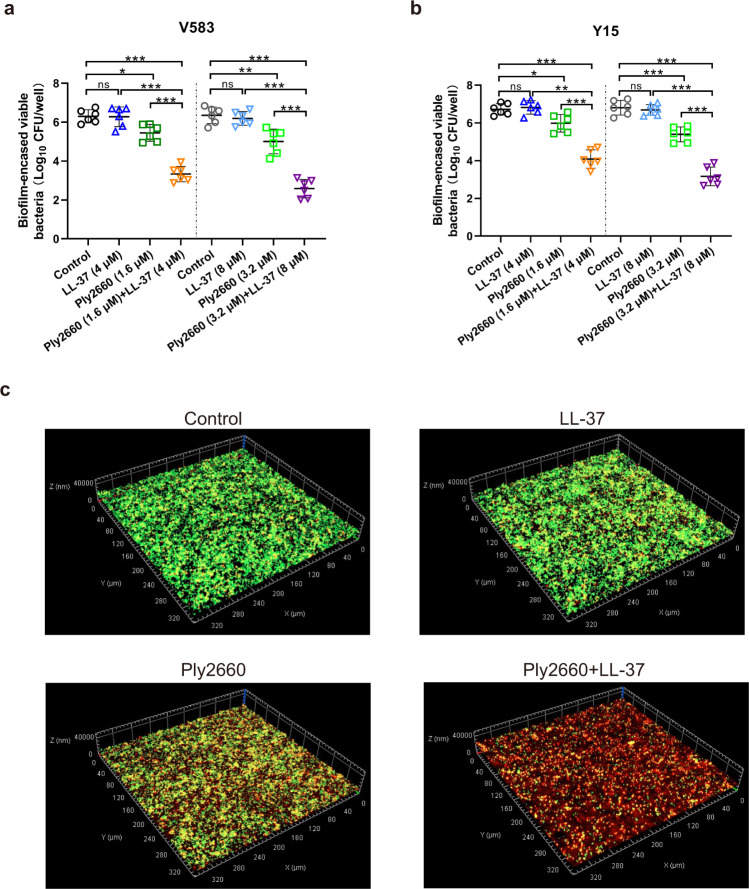
Treatment of preformed biofilms and bacterial viability of LL-37 and/or Ply2660. Bactericidal activity of LL-37 and/or Ply2660 against preformed biofilms of *E. faecalis* isolates **a** V583 and **b** Y15. Biofilms were allowed to develop for 24 h and then treated with LL-37 and/or Ply2660 for 6 h at 37 °C. Bacteria without any treatment served as a control. Results are expressed as the number of viable bacteria [in log_10_ CFU/well]. The data are presented as the means ± SD from two independent assays with three biological replicates in each assay; error bars represent standard deviation. Statistical significance was calculated using one-way ANOVA followed by Tukey’s multiple comparison test. *P < 0.05; **P < 0.01; ***P < 0.001; ns, not significant. **c** Representative confocal microscope images of LIVE/DEAD-stained *E. faecalis* Y15 in 24-h-established biofilms after treatment with the indicated concentrations of LL-37, Ply2660, or a mixture of both at 37 °C for 6 h.

Next, the abilities of LL-37 and/or Ply2660 against established (24 h) biofilms were investigated. As observed before, LL-37 alone had no effect on the number of viable bacteria in established biofilms of *E. faecalis* V583 and Y15 after 6 h exposure, and the effect of the Ply2660 on biofilm colony counts was less compared to those of the Ply2660+LL-37 combination (Fig. 5a, b). To better evaluate the effectiveness of the Ply2660–LL-37 combination on biofilm killing, mature biofilms were treated with LL-37 and/or Ply2660 and visualized by confocal laser scanning microscopy (CLSM) after the live/dead staining. SYTO 9 stains living bacterial cells with green fluorescence, and PI stains cells with impaired membranes with red fluorescence. As shown in Fig. 5c, after 24 h of incubation without treatment, *E. faecalis* Y15 displayed well-structured biofilms. LL-37 treatment did not show any obvious change compared to the untreated control. However, Ply2660 increased the number of dead bacteria in the biofilm. The combined treatment led to strikingly larger amounts of red-stained dead cells. These results demonstrated the synergistic effect of Ply2660 and LL-37 against *E. faecalis* biofilms. To determine whether LL-37 or Ply2660 directly affect biofilm extracellular matrix, *E. faecalis* Y15 biofilms after LL-37 and/or Ply2660 treatment were stained to visualize different components of the biofilm using Alexa Fluor 350-labeled Concanavalin A (polysaccharides) and SYPRO Ruby Biofilm Matrix stain (proteins) (Supplementary Fig. 3). LL-37 or Ply2660 alone, or their combination showed no obvious changes in fluorescence intensity (for either polysaccharides or protein respectively) from the CLSM images. These results suggested that the action of Ply2660 and LL-37 against *E. faecalis* biofilms is mainly due to the destruction of the biofilm-embedded cells.

### The combination of Ply2660 and LL-37 suppresses the dissemination of *E. faecalis* in infected mice

In light of the synergistic effect of the combination of Ply2660 and LL-37 on *E. faecalis* in vitro, we evaluated the therapeutic potential of this synergistic action. Before that, the hemolytic activities of LL-37 and Ply2660 against human red blood cells were determined as an indication of their toxicity toward mammalian cells. As shown in Supplementary Fig. 4, neither LL-37 nor Ply2660 exhibited notable hemolytic activity. Minor hemolytic effects (less than 13.5%) were observed for LL-37 at a concentration of 64 μM, whereas Ply2660 exhibited negligible hemolytic activity. To further evaluate the safety of LL-37 and Ply2660 in vivo, LL-37 and/or Ply2660 at the therapeutic dose were intraperitoneally injected into mice, and the changes in body weight were monitored for 14 days. The results showed that all mice from each group remained healthy, as indicated by the gradual increase in body weight during the observation period (Supplementary Fig. 5a). At 14 days after injection, lung, liver, kidney, and spleen were collected to observe whether or not these organs suffer from toxic damage. As shown in Supplementary Fig. 5b, LL-37 or Ply660 alone or the combination of both did not cause pathologic injuries.

**Fig. 6:**
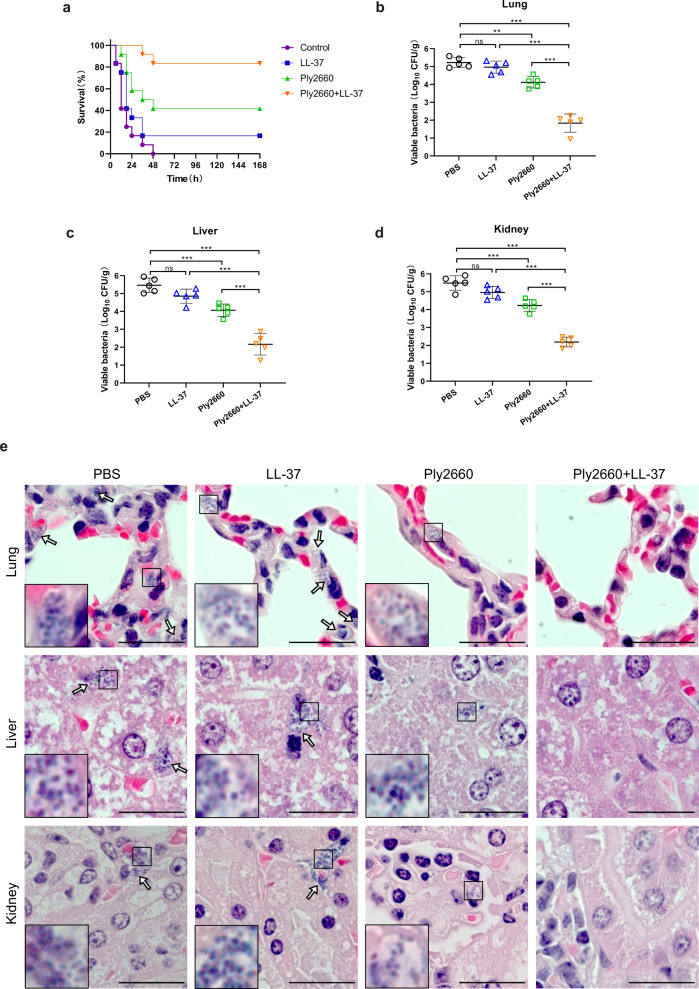
Bacterial loads in the organs of *E. faecalis*-infected mice treated with LL-37 and/or Ply2660. **a** Survival curves of mice infected intraperitoneally with *E. faecalis* V583 (high dose, 1.8 × 10^9^ CFU) and treated with a single dose of LL-37, Ply2660, a combination of LL-37 and Ply2660, or PBS via intraperitoneal injection (*n* = 12 for each group). For examination of bacterial loads in the **b** lung, **c** liver, and **d** kidney, mice were infected intraperitoneally with a lower dose (8 × 10^7^ CFU) of *E. faecalis* V583 and treated as above (*n* = 5 for each group). The bacterial load was calculated by plating the samples on BHI agar. The data are presented as the means ± SD from five mice; error bars represent the standard deviation. Statistical significance was calculated using one-way ANOVA followed by Tukey’s multiple comparison test. **P < 0.01; ***P < 0.001; ns, not significant. **e** Bacterial detection (arrows) in tissue sections with H&E staining. Scale bars, 20 μm.

Thereafter, the therapeutic effect of the Ply2660+LL-37 combination was evaluated in a murine model of peritoneal septicemia. Accordingly, BALB/c mice were infected with a high dose (1.8 × 10^9^ CFU) of *E. faecalis* V583 by intraperitoneal injection, and PBS, LL-37, Ply2660, or the Ply2660+LL-37 combination was administered 1 h post-infection. As shown in Fig. 6a, almost all mice in the PBS-treatment group died within 24 h. LL-37 barely had a therapeutic effect against intraperitoneal challenge with *E. faecalis*, whereas treatment with Ply2660 was able to rescue 58.3% of the infected mice. The combination of Ply2660 and LL-37 showed improved survival rates, rescuing 83.3% of the infected mice.

Next, BALB/c mice were infected with a lower dose (8 × 10^7^) of *E. faecalis* V583, and the treatment protocol was the same as before. After 24-h treatment, samples were collected from harvested organs for quantification of *E. faecalis*. As shown in Fig. 6b–d, high bacterial burdens were detected in the lung, liver, and kidney of mice in the PBS-treated group, indicating *E. faecalis* dissemination from the peritoneal cavity to multiple organs. Treatment with LL-37 alone did not cause a significant reduction in bacterial burden compared to the PBS-treated group. In contrast, treatment with Ply2660 reduced the bacterial burden in the lungs by approximately 1.1-log_10_ CFU compared to the PBS-treated group (Fig. 6b). A similar effect was observed in the liver and kidney (Fig. 6c, d). Moreover, the Ply2660+LL-37 combination caused a significant reduction in the bacterial burden compared to each treatment alone. Compared with the PBS-treated group, on average, a more than 3.0-log_10_ CFU reduction in bacterial burden was detected in the Ply2660+LL-37 group (Fig. 6b–d). As seen in Fig. 6e, a lot of coccus-shaped bacteria appeared in lung, liver, and kidney of mice from PBS-treated group and LL-37-treated group, whereas the number of such bacteria decreased in the organs of mice of the Ply2660-treated group, and few or no cocci were seen in those of mice of the Ply2660+LL-37 group. These results showed that the combination of Ply2660 and LL-37 demonstrated higher efficacy against *E. faecalis* dissemination than each treatment alone.

### The combination of Ply2660 and LL-37 attenuates organ injury in *E. faecalis*-infected mice

**Fig. 7:**
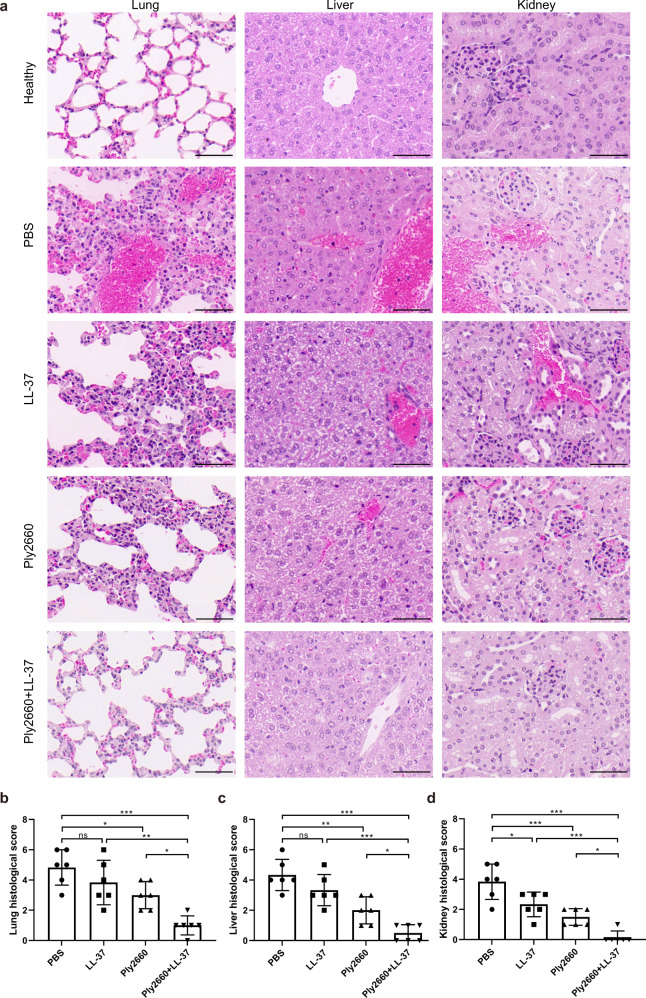
Histological evaluation of organ injuries in *E. faecalis*-infected mice treated with LL-37 and/or Ply2660. **a** Samples of the lung, liver, and kidney from mice with different treatments were fixed in 4% formalin, and tissue sections were prepared for H&E staining. Scale bars, 50 μm. Pathological scores of the **b** lung, **c** liver, and **d** kidney were evaluated from three random fields by two independent scientists. The data are presented as the means ± SD; error bars represent the standard deviation. Statistical significance was calculated using one-way ANOVA followed by Tukey’s multiple comparison test. *P < 0.05; **P < 0.01; ***P < 0.001; ns, not significant.

The therapeutic effects of LL-37 and/or Ply2660 were also confirmed by histological analysis. The hematoxylin and eosin (H&E)-stained sections (Fig. 7a) showed dramatic pathological damage in tissues of mice treated with PBS, including a high degree of inflammatory cell infiltration, severe blood stasis and hemorrhage, thickened respiratory membranes, hepatocyte degeneration and necrosis, hepatic sinusoidal dilation and congestion, renal tubular interstitial congestion with blurred brush border and clearly visible cast formation. However, LL-37 or Ply2660 treatment partially alleviated the tissue damage that occurred due to the *E. faecalis* infection. Remarkably, combined therapy with Ply2660+LL-37 almost completely abolished the inflammation damage induced by *E. faecalis* (Fig. 7a). Organ histological score analysis (Fig. 7b–d) confirmed these findings; the injury scores of lung, liver, and kidney from mice in the combined therapy group were significantly lower than those of the mice in the other groups, illustrating the contribution of the combination of Ply2660 and LL-37 to the restoration of *E. faecalis*-induced organ injury.

## Discussion

*Enterococcus faecalis* commonly causes difficult-to-treat infections because of intrinsic and acquired resistance to a wide range of antibiotics^4^. Especially during the COVID-19 pandemic, *E. faecalis* co-infection in hospitalized COVID-19 patients increased the risk of treatment failure and death^41,42^. Moreover, when linezolid and daptomycin received licensing for the treatment of infections caused by vancomycin-resistant enterococci, resistant isolates began to appear in clinical settings since then^43,44^. Novel alternative antimicrobial agents and therapeutic strategies are urgently needed to combat *E. faecalis* infections. In this context, antimicrobial peptides and phage endolysins are considered promising candidates, and efforts to achieve clinical application are currently accelerating.

*Enterococcus* *faecalis* is protected by a thick mesh of peptidoglycan that maintains cellular integrity^45^. Nevertheless, this bacterium can be attacked by phage endolysins, which are efficient enzymes that directly cleave covalent bonds within the cell wall peptidoglycan and eventually cause bacterial lysis and death^46^. The endolysin Ply2660 has attracted our attention because it has a cell wall-binding domain of SH3b similar to that of the endolysin PlyV12 from an enterococcal phage^29^, implying the probability of Ply2660 targeting *E. faecalis*. In this study, the phage endolysin Ply2660 was found to degrade the peptidoglycan of *E. faecalis* and, thus, kill this bacterium. In addition, Ply2660 also exhibited lytic activities to vancomycin-resistant isolates, which are responsible for nosocomial infections to some extent. However, Ply2660 did not kill *E. faecium* isolates. As far as we know, several of the so far reported *E. faecalis* endolysins could also lyse *E. faecium*, such as IME-EF1^47^, PlyV12^29^, and ORF9 of phage ΦEF24c^48^. However, the endolysin LysEF-P10 derived from an *E. faecalis* phage also did not kill *E. faecium*^49^. The peptidoglycan compositions of *E. faecalis and E. faecium* show minor differences. They have the same pentapeptide stem linked to the *N*-acetylmuramic acid but differ in the cross-linking bridge. The bridge is L-Ala-L-Ala in the peptidoglycan of *E. faecalis*, but D-Asp in that of *E. faecium*^50–52^, which may be the reason why *E. faecium* cannot be killed by Ply2660. Ply2660 possesses a CHAP catalytic domain, which can serve as *N*-acetylmuramoyl-L-alanine amidase or endopeptidase^53^. Since the cleavage site of *N*-acetylmuramoyl-L-alanine amidase is the same in *E. faecalis* and *E. faecium*, it is very likely that Ply2660 exerted endopeptidase activity, leading to its different bactericidal ability against *E. faecalis* and *E. faecium*, but this needs to be further ascertained.

**Fig. 8:**
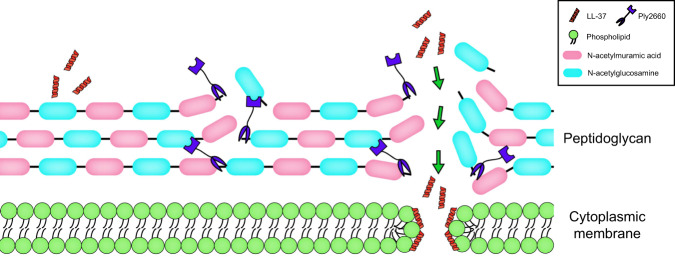
Schematic representation of the lysis of *E. faecalis* by the phage endolysin Ply2660 and the antimicrobial peptide LL-37. *Enterococcus faecalis* is protected against LL-37 by a thick mesh of peptidoglycan. However, Ply2660 degrades the cell wall peptidoglycan of *E. faecalis*, making the cytoplasmic membrane more accessible to LL-37. Subsequently, LL-37 interacts with the cytoplasmic membrane and inserts itself into lipid bilayers, leading to pore formation in the lipid membranes and lysis of *E. faecalis*. Thus, the combination of Ply2660 and LL-37 exerts synergistic antimicrobial effects against *E. faecalis*.

It is well documented that the human antimicrobial peptide LL-37 exerts bactericidal activity against a variety of bacteria^20,39^. However, the findings in our study indicated that *E. faecalis* could resist the killing by LL-37. Moreover, *E. faecalis* is likely to be resistant not just to LL-37, but also to other antimicrobial peptides of host or bacterial origin. A previous study revealed that *E. faecalis* displays high intrinsic resistance against bacitracin, an antimicrobial peptide isolated from a strain of *Bacillus*^54^. It has been reported that *E. faecalis* gelatinase can cleave LL-37, which may be an important strategy exploited by *E. faecalis* to resist the killing of antimicrobial peptides^55^. In addition, we speculated whether some so far undiscovered surface-associated proteins of *E. faecalis* might hinder the access of antimicrobial peptides to the cytoplasmic membrane. Similarly, the surface-associated M1 protein of *Streptococcus pyogenes* has been demonstrated to sequester and neutralize LL-37 antimicrobial activity through its N-terminal domain^56^. However, additional studies are required to ascertain whether a similar mechanism applies to *E. faecalis*. From the perspective of pathogen-host interactions, the resistance of *E. faecalis* against antimicrobial peptides might be important for the successful invasion of this bacterium into the human host. In this study, we showed that although LL-37 alone did not kill *E. faecalis*, the addition of Ply2660 could trigger the antibacterial activity of LL-37 against *E. faecalis*, and the combination of both showed high efficacy at low concentrations, which might encourage their combined use for the control of *E. faecalis*. The synergy between Ply2660 and LL-37 could be explained as follows: degradation of the cell wall by Ply2660 makes the cytoplasmic membrane of *E. faecalis* more accessible to LL-37 (Fig. 8).

The ability of *E. faecalis* to form biofilms can exacerbate the pathogenicity that leads to life-threatening infections. Furthermore, when biofilms are produced by multidrug-resistant *E. faecalis*, the chances of successfully eradicating them are even smaller^13^. Previous studies have demonstrated that phage therapy was more effective in some cases for combating *E. faecalis* than conventional antibiotics, particularly when it comes to infections with multidrug-resistant biofilm-producing isolates^57,58^. Moreover, several phage endolysins, such as lys08 and ϕEf11 ORF28, showed antibiofilm activity against *E. faecalis* biofilms^59,60^. Similar to these previous studies, Ply2660 also exhibited antibiofilm activity against *E. faecalis* biofilms to some extent. Compared to the single Ply2660 treatment, the Ply2660+LL-37 combination showed distinctly higher antibiofilm activity against *E. faecalis* biofilms. Notably, Ply2660 and LL-37 did not destroy the main component of the biofilm extracellular matrix. The ability of Ply2660+LL-37 combination to inhibit biofilm formation is most likely attributable to their powerful bactericidal activity before biofilm formation. In addition, the Ply2660+LL-37 combination also exhibited excellent bactericidal efficacy in established biofilms. By adopting a biofilm lifestyle, bacteria grow slowly or do not grow due to a deficiency of nutrients that results in tolerance to antibiotics^14^. The mode of action of Ply2660 and LL-37, which is unlike antibiotics, does not require the metabolic machinery of the cell as they target the peptidoglycan and cytoplasmic membrane. Thus, the Ply2660+LL-37 combination could kill dormant cells in biofilm as well as planktonic cells. Furthermore, since both endolysins and antimicrobial peptides have low molecular weight, it is probable that they can penetrate more easily into deeper layers of the biofilm. It has been reported previously that the catalytic domain of an endolysin can help it penetrate into the biofilm^61^.

Combined therapy offers a promising tool to cope with the rising global crisis of antibiotic resistance^62^. Phage endolysins and antimicrobial peptides have been found to act synergistically in the treatment of infections due to antibiotic-resistant bacteria^38,63^. The combined application of phage endolysins and antimicrobial peptides against antibiotic-resistant bacteria is an interesting research area that is still in its infancy^38^. In this study, we showed that the combination of Ply2660 and LL-37 not only displayed synergistic activity against *E. faecalis* in vitro but also elicited synergistic effects in vivo, increasing the survival rate, reducing the bacterial burden and alleviating tissue inflammation in *E. faecalis*-infected mice. Despite our study verifying the therapeutic effect only in the *E. faecalis*-induced murine model of peritoneal septicemia, the potential of the Ply2660+LL-37 combination is clear. This combination may be able to treat intra-abdominal and pelvic infections. However, it is necessary to pay attention to the possibility of an immune response caused by Ply2660 after systemic administration, and further studies involving its clinical application need to be carried out. In addition, it also remains to be established whether the combined Ply2660 and LL-37 could be coated on the catheter surfaces to avoid catheter-related infections, which are of major relevance among *E. faecalis* infections.

Natural antimicrobial peptides often have cytotoxic effects, which limit their clinical application. Although LL-37 has entered the phase II clinical trial^64^, it indeed shows minor hemolytic activity at a high concentration, but its therapeutic dose in this study did not cause toxicity in mice. Although Ply2660 does not exhibit hemolytic activity in vitro, its possible immunogenicity cannot be excluded. Combination therapy may be a suitable strategy to fight *E. faecalis* infection in vivo by maximizing effectiveness and minimizing the side effects of Ply2660 and LL-37. On the one hand, the high production cost is another limitation of the clinical application of endolysins and antimicrobial peptides. To reduce production costs, future studies should consider further increasing the solubility and expression level of endolysins, as well as choosing more cost-effective expression systems, or developing prodrugs fusing the endolysins and antimicrobial peptides. Our ongoing research is also designing new antimicrobial peptides with smaller molecular weights to reduce costs, improve antibacterial activity, and reduce toxicity. Thereby, we expect to find safer antimicrobial peptides for optimized combinations.

In conclusion, this work shows the potential of the synergistic interaction between the phage endolysin Ply2660 and the antimicrobial peptide LL-37 to develop a more efficient antimicrobial combination therapy against infections caused by vancomycin-resistant and multidrug-resistant *E. faecalis*. The high efficacy of this combination therapy is based on the mode of action of Ply2660-induced cell wall degradation and subsequent LL-37-mediated membrane damage. Overall, this strategy provides a promising antibiotic-free alternative approach to combat drug-resistant bacteria and opens a new path for the treatment of life-threatening bacterial diseases. Further studies are needed to exploit the potential of this combination for optimizing health outcomes.

## Methods

### Bacterial strains and growth conditions

The *E. faecalis* isolates used in the present study were the reference strain ATCC 29212, the vancomycin-resistant reference strain ATCC 51299^65^, the vancomycin-resistant clinical isolate V583^66^, the multidrug-resistant clinical isolate SY-1^67^, and the Y15 isolate (resistance to erythromycin, gentamicin, and streptomycin) isolated from bovine feces with the capacity of producing biofilm in this study. The *E. faecium* isolates used in the present study were the vancomycin-resistant reference strain ATCC 700221, the vancomycin-resistant EFM30 isolate, and the vancomycin-susceptible EFM171 isolate, which were isolated from bovine feces in this study. All *E. faecalis* and *E. faecium* isolates were grown in Trypticase soy broth (TSB), BHI broth, or BHI agar (Difco, Detroit, MI, USA) at 37 °C.

### Bioinformatics analysis

The 3D structure of LL-37 was obtained from the RCSB Protein Data Bank^68^. Protein domains of Ply2660 were identified using SMART^69^. Protein structure homology modeling of Ply2660 was conducted by SWISS-MODEL^70^. The structures of LL-37 and Ply2660 were presented using PyMOL (DeLano Scientific, San Carlos, CA, USA).

### Protein expression and purification

The *ply2660* gene (GenBank accession number CP031379.1) was chemically synthesized, and inserted into the pET22b plasmid (Novagen, San Diego, CA, USA) by BGI Biotech (Beijing, China). The recombinant plasmid pET22b-ply2660 was sequenced for confirmatory reasons and transformed into competent *Escherichia coli* BL21 (DE3) cells. Protein expression was induced by 0.5 mM of isopropyl 1-thio-β-D-galactopyranoside (Sigma-Aldrich, St. Louis, MO, USA) with shaking for 12 h at 16 °C. The cultures were then centrifuged, and the cell pellets were resuspended in binding buffer (20 mm Tris-HCl, pH 7.9, 0.5 m NaCl, and 20 mm imidazole). Then, the cells were disrupted by sonication, and supernatants were collected by centrifugation at 10,000×*g* for 10 min. His-tagged protein was purified using a Ni-Sepharose^TM^ 6 Fast Flow column (GE Healthcare, Uppsala, Sweden) according to the manufacturer’s instructions. The elution buffer was changed to PBS using a PD-10 desalting column (GE Healthcare, Uppsala, Sweden). The purified protein was analyzed using 12% SDS-PAGE. All gels derive from the same experiment and were processed in parallel. The protein concentration was determined using a bicinchoninic acid protein assay kit (Beyotime, Shanghai, China).

### Determination of minimal inhibitory concentration

The MICs of LL-37 and Ply2660 were determined using the standard broth microdilution method recommended by the Clinical and Laboratory Standards Institute^71^. Briefly, a final bacterial suspension of 5 × 10^5^ CFU/ml in cation-adjusted Mueller–Hinton broth (Becton Dickinson, Sparks, MD, USA) was exposed to LL-37 or Ply2660 in a series of twofold serial dilutions in 96-well polypropylene microtiter plates (Nest, Wuxi, China). Cells were challenged with 0.5–256 μM of LL-37, or 0.2–51.2 μM of Ply2660 in triplicate. The MIC value was defined as the lowest concentration of LL-37 or Ply2660 that inhibited visible growth.

### Bactericidal assays

*Enterococcus faecalis* isolates were grown in BHI broth at 37 °C to an optical density at 600 nm (OD_600_) of 0.8. Each isolate was harvested and diluted in PBS (pH 7.4) to a concentration of 2 × 10^6^ CFU/ml. Then, 25 μl of bacterial suspensions (5 × 10^4^ CFU) were added into the 96-well polypropylene microtiter plates (Nest, Wuxi, China), and incubated in the presence of 4 μM of LL-37 (GL peptide Inc., Shanghai, China, purity > 98%), 1.6 μM of Ply2660, or a mixture of both in a total volume of 200 μl PBS. As an untreated control, bacteria were incubated in PBS. After incubation for 1 h at 37 °C, each sample was serially diluted, and 10 μl aliquots of the serial dilutions were spotted onto BHI agar. The plates were incubated at 37 °C for 18 h.

For time-kill experiments, *E. faecalis* V583 (1 × 10^5^ CFU) was incubated in the presence of 4 μM of LL-37, 1.6 μM of Ply2660, or a mixture of both in PBS in polypropylene tubes in a total volume of 500 μl. After incubation at 37 °C for 30 to 120 min, each sample was serially diluted, and 100 μl aliquots of the serial dilutions were plated on BHI agar for CFU counting. For concentration-kill experiments, *E. faecalis* V583 (1 × 10^5^ CFU) was incubated in the presence of 2–8 μM of LL-37, 0.8–3.2 μM of Ply2660, or a mixture of both in a total volume of 500 μl at 37 °C for 60 min. Aliquots were serial diluted and plated to assess viability.

*Enterococcus faecium* isolates (1 × 10^5^ CFU) were incubated in the presence of 3.2 μM of Ply2660 in a total volume of 500 μl at 37 °C for 60 min. Aliquots were serial diluted and plated to assess viability.

### Transmission electron microscopy

*Enterococcus faecalis* V583 was cultivated in 5 ml of BHI broth at 37 °C until the mid-logarithmic phase. The cultures were washed three times and resuspended in PBS at a concentration of 5 × 10^8^ CFU/ml, and exposed to 4 μM of LL-37, 1.6 μM of Ply2660, or a mixture of both at 37 °C. Bacteria without any treatment were used as control. After 1 h, the samples were fixed with 2.5% glutaraldehyde overnight and then post-fixed with 2% osmic acid at 4 °C for 2 h. Following dehydration in upgraded ethanol, the samples were embedded in SPI-Pon 812 resin. Polymerization was done at 70 °C for 2 days, and 65–70-nm sections were cut using a UC6 ultra-microtome (Leica Microsystems, Vienna, Austria) and post-stained with uranyl acetate and lead citrate. Electron micrographs were visualized by an H-7650 transmission electron microscope (Hitachi, Tokyo, Japan).

### Cell wall degradation assay

The effect of LL-37 and/or Ply2660 on the degradation of *E. faecalis* cell walls was examined by measuring the turbidity of the bacterial suspension. *Enterococcus faecalis* strain V583 was grown in 500 ml of BHI broth at 37 °C to the mid-logarithmic phase and washed three times with PBS. The bacterial pellet was resuspended in 14 ml of 4% SDS and boiled for 30 min. The cell wall was isolated by centrifugation at 10,000 × *g* for 15 min, washed four times with distilled water, and then incubated in 10 ml of buffer (0.5 mg/ml trypsin in 0.1 M Tris-HCl, pH 6.8, and 20 mM CaCl_2_) at 37 °C for 18 h. Thereafter, the sample was washed four times with distilled water, resuspended in 2 ml 10% trichloroacetic acid, and incubated at 4 °C for 6 h. The cell wall was further washed four times with distilled water and resuspended in PBS. The purified *E. faecalis* cell wall suspension was incubated in the presence of 4 μM of LL-37, 1.6 μM of Ply2660, or a mixture of both at 37 °C. The cell wall-degrading enzyme mutanolysin (Sigma-Aldrich, St. Louis, MO, USA) was used as a positive control. The OD_600_ was monitored for 5 h at 1-h intervals.

### Detection of membrane permeability

Bacterial cell membrane damage induced by the different treatments was monitored by detecting PI uptake. Briefly, mid-logarithmic phase cultures of *E. faecalis* strain V583 were resuspended in PBS to 1 × 10^6^ CFU/ml and incubated in the presence of 4 μM of LL-37, 1.6 μM of Ply2660, or a mixture of both at 37 °C for 1 h. Then, 10 μl PI (Invitrogen, Eugene, OR, USA) was added to a final concentration of 4 μg/ml, and samples were incubated for 20 min at 25 °C. Fluorescence was detected using an EnSpire Multiscan Spectrum (PerkinElmer, Massachusetts, USA), at an excitation wavelength of 535 nm and an emission wavelength of 617 nm.

### Inhibition of biofilm formation

Mid-logarithmic growth-phase cultures of *E. faecalis* V583 and Y15 were diluted in TSB medium to 1 × 10^8^ CFU/ml. Fifty microliters of this bacterial suspension were exposed to the various concentrations of LL-37 and/or Ply2660, then added to 96-well polypropylene microtiter plates (Corning, NY, USA). Bacteria without any treatment served as a control. After 24-h incubation at 37 °C, planktonic bacteria were washed from the biofilms. The biofilms were stained with 1% crystal violet (Sigma-Aldrich, St. Louis, MO, USA) for 20 min, washed, and solubilized with 33% acetic acid. The optical density at 600 nm was determined by a microplate spectrophotometer. Minimum biofilm inhibitory concentration (MBIC) is defined as the lowest concentration at which there were no detectable biofilms after 24-h incubation^72^. The number of viable bacteria in biofilms exposed to the indicated concentrations of LL-37 and/or Ply2660 was also quantified by plate colony counts.

### Treatment of established biofilm

Mid-logarithmic growth-phase cultures of *E. faecalis* V583 and Y15 were diluted and added to 96-well polypropylene microtiter plates (Corning, NY, USA) without treatment and incubated for 24 h at 37 °C to allow biofilm formation. Afterward, planktonic bacteria were removed, and the biofilms were gently washed twice with PBS. Thereafter, the adhered biofilms were treated with the indicated concentrations of LL-37 and/or Ply2660. Bacteria without any treatment were used as a control. After 6-h incubation at 37 °C, the medium was removed, and the biofilm was gently washed three times with PBS. To assess the biofilm-killing efficacy of the different treatments, the number of viable bacteria in the biofilm was quantified by plate colony counts.

For confocal microscopy analysis, 24-h-established biofilms were formed by inoculating 2 ml of an *E. faecalis* Y15 cell suspension containing approximately 2 × 10^6^ CFU/ml in glass-bottom microwell dishes (Nest, Wuxi, China). After washing twice, biofilms were exposed to 4 μM of LL-37, 1.6 μM of Ply2660, or a mixture of both for 6 h at 37 °C. At the end of the treatment, biofilms were washed twice with PBS and stained with the Live/Dead® BacLightTM kit (Invitrogen, Eugene, OR, USA). For exopolysaccharide visualization, biofilms were stained with Alexa Fluor 350-labeled Concanavalin A (Invitrogen, Eugene, OR, USA). The proteins in the biofilms were stained with SYPRO Ruby Biofilm Matrix stain (Invitrogen, Eugene, OR, USA). Samples were visualized using a confocal scanning laser microscope (Zeiss LSM 880, Jena, Germany).

### Hemolysis assay

Human red blood cell suspensions (4% (w/v)) in PBS (pH 7.4) were incubated with various concentrations of LL-37 or Ply2660 for 1 h at 37 °C. Following this, the samples were centrifuged at 500 × *g* for 10 min, and the supernatant absorbance was read at 415 nm. A 1% Triton X-100 (Sigma-Aldrich, St. Louis, MO, USA) solution served as positive Control (A_triton_). The percentage of hemolysis was calculated using the following formula: hemolysis % = [(A_sample_ − A_PBS_) / (A_triton_ − A_PBS_)] × 100.

### Toxicity to BALB/c mice

All of the experiments involving mice were conducted in accordance with the guidelines and policies of Laboratory Animals of the Ministry of Science and Technology of China. The protocols were reviewed and approved by the Animal Ethics Committee of the Harbin Veterinary Research Institute of the Chinese Academy of Agricultural Sciences (200811-03). Six-week-old specific pathogen-free BALB/c mice (Vital River, Beijing, China) were divided into four groups, with five mice in each group. LL-37 (10 mg/kg), Ply2660 (16 mg/kg), a combination of LL-37 (10 mg/kg) and Ply2660 (16 mg/kg), or PBS as control was injected intraperitoneally into mice. The mice were weighed every 24 h for 14 days. Then, the mice were euthanized, and the organs were collected for observation of gross lesions change.

### Combined therapy in the murine model of peritoneal septicemia

Six-week-old specific pathogen-free BALB/c mice (Vital River, Beijing, China) were injected intraperitoneally with 8 × 10^7^ CFU of *E. faecalis* strain V583 suspended in 100 μl of BHI broth with 6% mucin. The treatment experiment was performed 1 h after the *E. faecalis* challenge. The infected mice were divided into four groups, with five mice in each group. LL-37 (10 mg/kg), Ply2660 (16 mg/kg), a combination of LL-37 (10 mg/kg) and Ply2660 (16 mg/kg), or PBS was injected intraperitoneally into mice. The mice were euthanized after 24-h treatment, and lung, liver, and kidney of each mouse were harvested, weighed, homogenized, and plated on agar for CFU counting. For histopathological analysis, lung, liver, and kidney samples were fixed in 4% formalin. After paraffin embedding, 4-μm tissue sections were stained with H&E according to the standard protocol and examined by light microscopy. Pathological scores based on the severity of hemorrhages and inflammation were respectively assigned as follows: normal (score = 0), minimal (<5% of focal areas; score = 1), mild (5–10% of focal areas; score = 2), moderate(11–30% of focal areas; score = 3), or severe (>30% of focal areas; score = 4). To determine the efficacy of LL-37 and/or Ply2660 on the survival rate of mice, an additional experiment was performed by injecting intraperitoneally a high dose (1.8 × 10^9^ CFU) of *E. faecalis* V583, and the following treatment protocol was the same as before, but with 12 mice in each group. The survival rate for each experimental group was monitored every 6 h for the first 24 h and then every 12 h for 7 days.

### Statistical analysis

Statistical analysis was conducted and graphs were generated using GraphPad Prism version 9.0 (GraphPad Software). The number of replicates is given in the respective figure legends. Statistical significance was analyzed using one-way analysis of variance (ANOVA), two-way ANOVA, Brown–Forsythe and Welch ANOVA, or unpaired *t*-tests. *P*-values of less than 0.05 were considered to be statistically significant.

## Supplementary information

Supplementary information

## Data Availability

All data supporting the findings of this study are included in the article and its Supplementary file. Additional data are available from the corresponding authors upon request.
