# Supplementary material for: Bactericidal synergism between phage endolysin Ply2660 and cathelicidin LL-37 against vancomycin-resistant Enterococcus faecalis biofilms
Source: NPJ Biofilms Microbiomes. 2023 Apr 6;9:16. doi: 10.1038/s41522-023-00385-5 (PMC10078070; doi:10.1038/s41522-023-00385-5)
Supplement: Supplementary file 1 — Supplementary information [file 41522_2023_385_MOESM1_ESM.pdf]

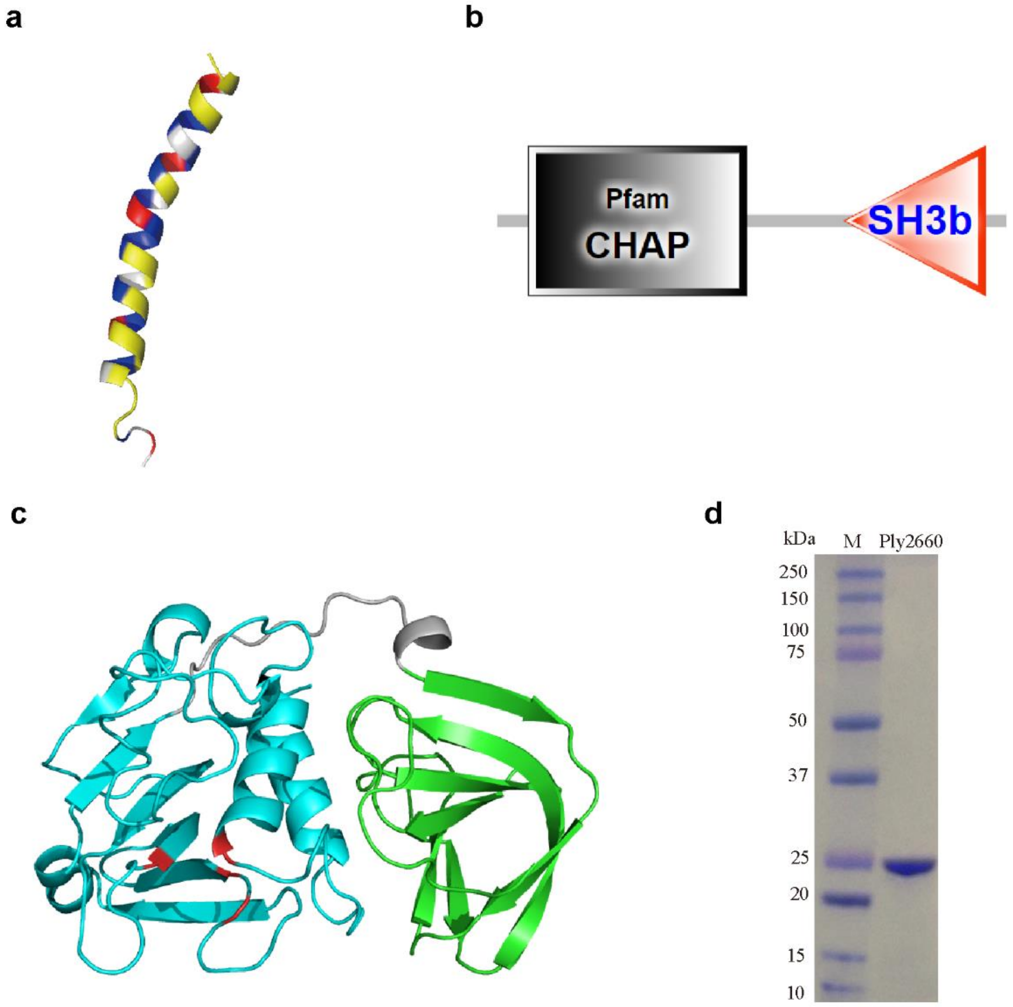

Supplementary Figure 1. General features of LL-37 and Ply2660.

(a) 3D model of LL-37. Basic, acidic, hydrophobic, and polar amino acids are shown as blue, red, yellow, and white, respectively. The 3D structures of LL-37 (PDB: 2K6O) were obtained from the Protein Data Bank. (b) Protein domains of Ply2660 identified by SMART. (c) 3D model of Ply2660. The N-terminal cysteine–histidine-dependent amidohydrolase/peptidase (CHAP) domain is shown in blue, the C-terminal SH3b domain is shown in green, and active-site residues (Cys26, His102, Glu118, and Asn120) are shown in red. Protein structure homology modeling was conducted by SWISS-MODEL using the 3D structure of phage lysin Ly7917 (PDB:5d74) as a template. Results are presented by PyMOL. (d) SDS-PAGE gel picture showing the purified Ply2660 expressed in *E. coli*. M, protein standards.

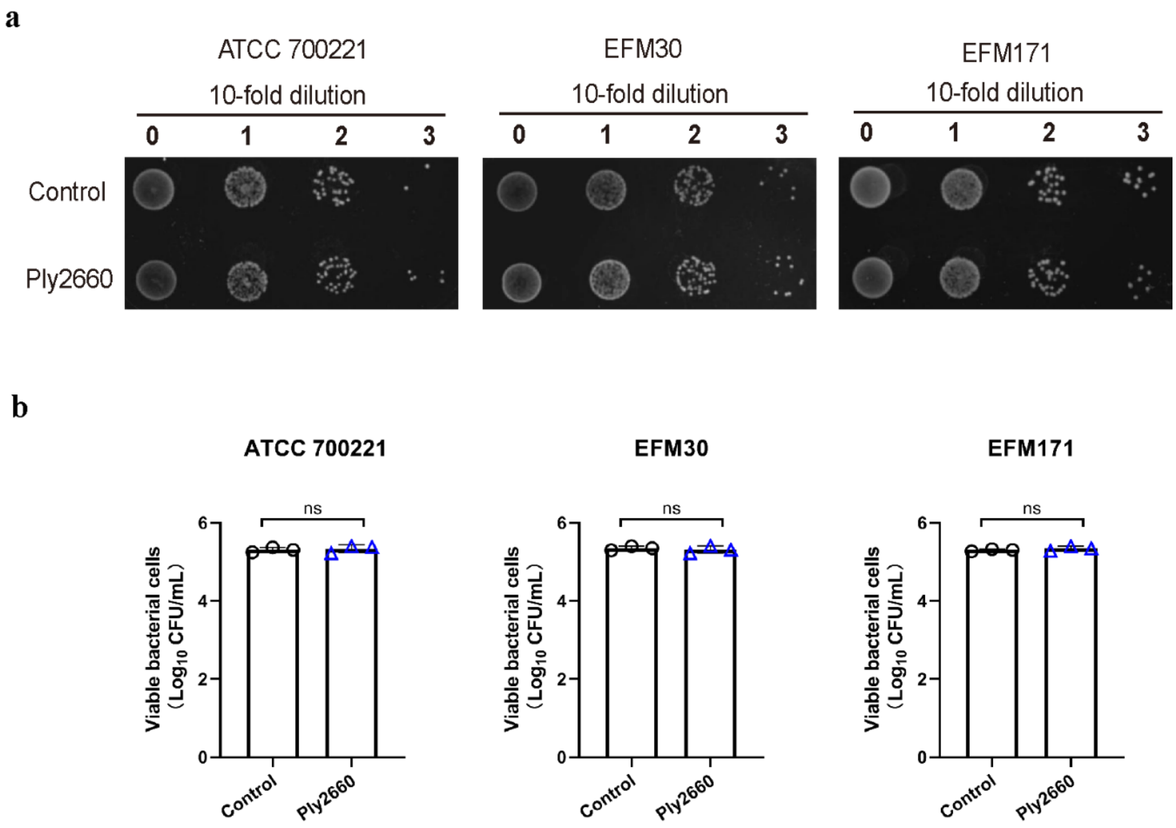

Supplementary Figure 2. Bactericidal activity of Ply2660 against *E. faecium* strains.

E. faecium strains were treated with 3.2 μM of Ply2660 and incubated for 1 h at 37°C. (a) Ten-fold serial dilutions of each sample were spotted onto BHI agar and incubated at 37°C for 18 h. Controls were treated with PBS alone. These assays were repeated three times at independent occasions with similar results, and representative experiments are shown. (b) Ten-fold serial dilutions of each sample were plated on BHI agar for calculation of viable bacteria. The data are presented as the means ± SD from three independent assays, error bars represent standard deviation. Statistical significance was calculated using unpaired *t* tests. ns, not significant.

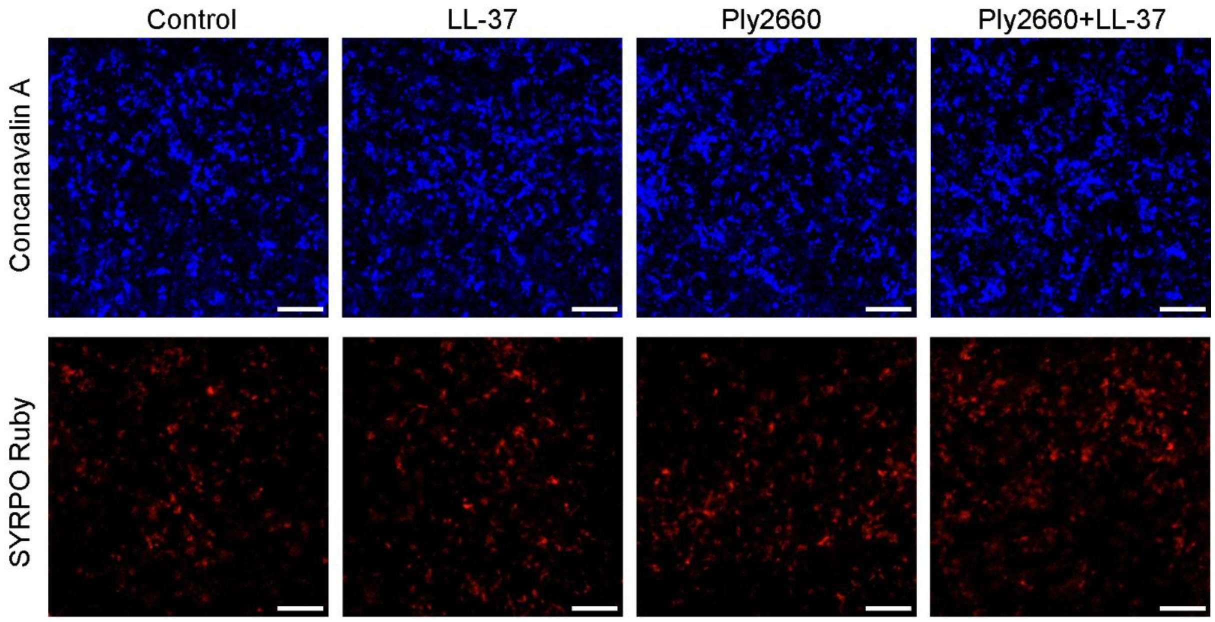

Supplementary Figure 3. Effect of LL-37 and Ply2660 on biofilm matrix components of *E. faecalis*.

CLSM imaging of *E. faecalis* (Y15) biofilms stained with Alexa Fluor 350-labelled Concanavalin A (polysaccharides, blue) and SYPRO Ruby Biofilm Matrix Stain (proteins, red) in 24-h-established biofilms of *E. faecalis* Y15 followed by 6 h treatment of LL-37 and/or Ply2660. Scale bar, 10 μm.

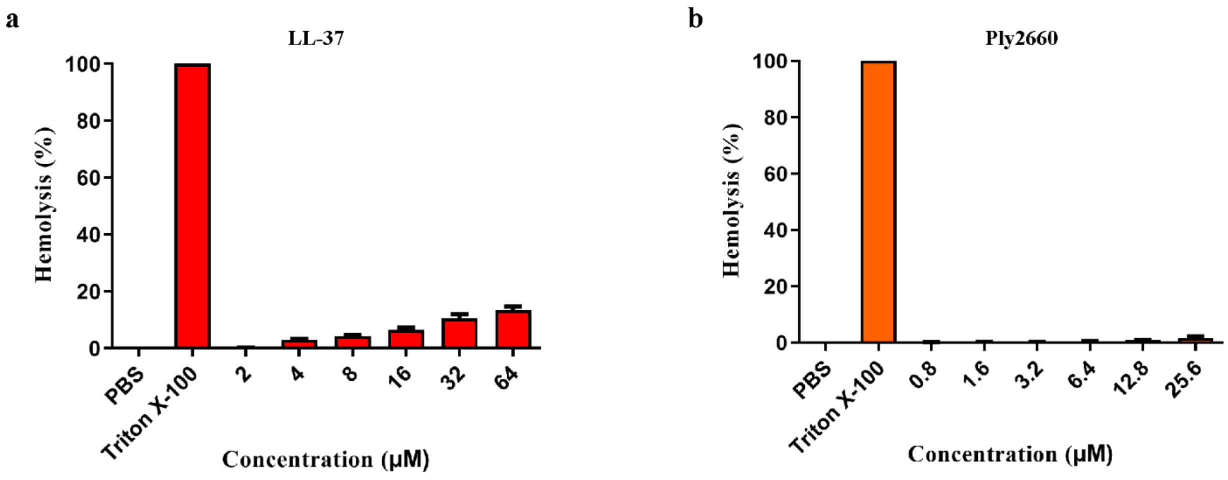

Supplementary Figure 4. Hemolytic activity of LL-37 and Ply2660.

Potential hemolytic activities of (a) LL-37 (2-64 μM) and (b) Ply2660 (0.8-25.6 μM) on human red blood cells. The data are presented as the means ± SD from three independent assays, error bars represent standard deviation. PBS served as negative control, Triton X-100 as positive control.

a

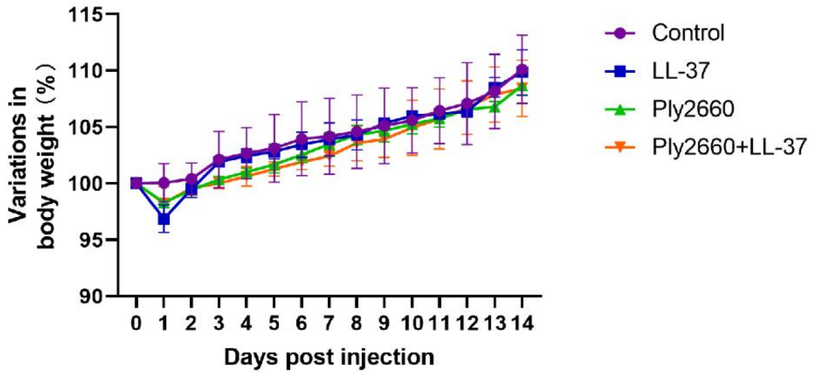

b

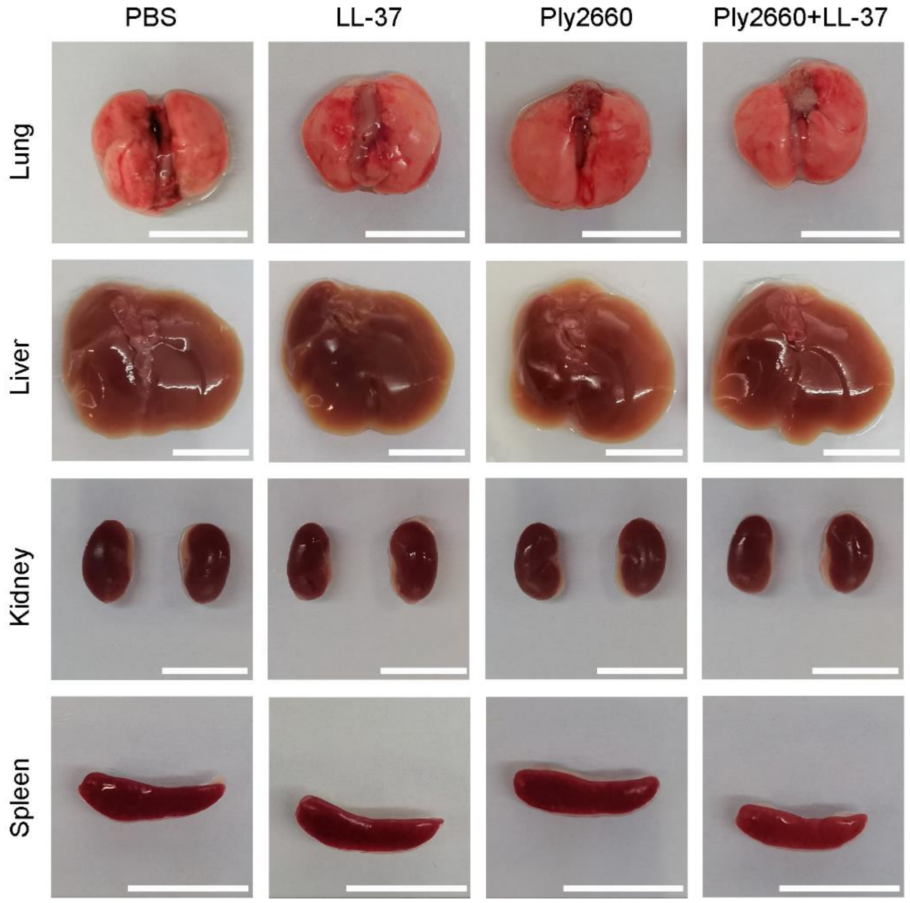

Supplementary Figure 5. Toxicity of LL-37 and Ply2660 *in vivo*.

(a) The body weights of five mice in each group were observed over 14 days after intraperitoneally injected with the LL-37 or/and Ply2660. The data are presented as the means ± SD, error bars represent standard deviation. (b) Organs of mice harvested after treatment with LL-37 or/and Ply2660. Scale bars, 10 mm. No gross lesions changes were seen in lungs, livers, kidneys, and spleens of mice from each group.
